# Financial Conflicts of Interest and Reporting Bias Regarding the Association between Sugar-Sweetened Beverages and Weight Gain: A Systematic Review of Systematic Reviews

**DOI:** 10.1371/journal.pmed.1001578

**Published:** 2013-12-31

**Authors:** Maira Bes-Rastrollo, Matthias B. Schulze, Miguel Ruiz-Canela, Miguel A. Martinez-Gonzalez

**Affiliations:** 1 Department of Preventive Medicine and Public Health, University of Navarra, Pamplona, Spain; 2 Departament of Molecular Epidemiology, German Institute of Human Nutrition Potsdam-Rehbruecke, Nuthetal, Germany; 3 CIBERobn, Instituto de Salud Carlos III, Madrid, Spain; University of Oxford, United Kingdom

## Abstract

**Background:**

Industry sponsors' financial interests might bias the conclusions of scientific research. We examined whether financial industry funding or the disclosure of potential conflicts of interest influenced the results of published systematic reviews (SRs) conducted in the field of sugar-sweetened beverages (SSBs) and weight gain or obesity.

**Methods and Findings:**

We conducted a search of the PubMed, Cochrane Library, and Scopus databases to identify published SRs from the inception of the databases to August 31, 2013, on the association between SSB consumption and weight gain or obesity. SR conclusions were independently classified by two researchers into two groups: those that found a positive association and those that did not. These two reviewers were blinded with respect to the stated source of funding and the disclosure of conflicts of interest.

We identified 17 SRs (with 18 conclusions). In six of the SRs a financial conflict of interest with some food industry was disclosed. Among those reviews without any reported conflict of interest, 83.3% of the conclusions (10/12) were that SSB consumption could be a potential risk factor for weight gain. In contrast, the same percentage of conclusions, 83.3% (5/6), of those SRs disclosing some financial conflict of interest with the food industry were that the scientific evidence was insufficient to support a positive association between SSB consumption and weight gain or obesity. Those reviews with conflicts of interest were five times more likely to present a conclusion of no positive association than those without them (relative risk: 5.0, 95% CI: 1.3–19.3).

An important limitation of this study is the impossibility of ruling out the existence of publication bias among those studies not declaring any conflict of interest. However, the best large randomized trials also support a direct association between SSB consumption and weight gain or obesity.

**Conclusions:**

Financial conflicts of interest may bias conclusions from SRs on SSB consumption and weight gain or obesity.

*Please see later in the article for the Editors' Summary*

## Abstract 2

Maira Bes-Rastrollo and colleagues examine whether financial conflicts of interest are likely to bias conclusions from systematic reviews that investigate the relationship between sugar-sweetened beverages and weight gain or obesity.

*Please see later in the article for the Editors' Summary*

## Editors' Summary

**Background:**

In our daily lives, we frequently rely on the results of scientific research to make decisions about our health. If we are healthy, we may seek out scientific advice about how much exercise to do to reduce our risk of a heart attack, and we may follow dietary advice issued by public health bodies to help us maintain a healthy weight. If we are ill, we expect our treatment to be based on the results of clinical trials and other studies. We assume that the scientific research that underlies our decisions about health-related issues is unbiased and accurate. However, there is increasing evidence that the conclusions of industry-sponsored scientific research are sometimes biased. So, for example, reports of drug trials sponsored by pharmaceutical companies sometimes emphasize the positive results of trials and “hide” unwanted side effects deep within the report or omit them altogether.

**Why Was This Study Done?:**

Although the effects of company sponsors on the conclusions of pharmaceutical research have been extensively examined, little is known about the effects of industry sponsorship on nutrition research, even though large commercial entities are increasingly involved in global food and drink production. It is important to know whether the scientific evidence about nutrition is free of bias because biased information might negatively affect the health of entire populations. Moreover, scientific evidence from nutrition research underlies the formulation of governmental dietary guidelines and food-related public health interventions. In this systematic review, the researchers investigate whether the disclosure of potential financial conflicts of interest (for example, research funding by a beverage company) has influenced the results of systematic reviews undertaken to examine the association between the consumption of highly lucrative sugar-sweetened beverages (SSBs) and weight gain or obesity. Systematic reviews identify all the research on a given topic using predefined criteria. In an ideal world, systematic reviews provide access to all the available evidence on specific exposure–disease associations, but publication bias related to authors' conflicts of interest may affect the reliability of the conclusions of such studies.

**What Did the Researchers Do and Find?:**

The researchers identified 18 conclusions from 17 systematic reviews that had investigated the association between SSB consumption and weight gain or obesity. In six of these reviews, a financial conflict of interest with a food industry was disclosed. Among the reviews that reported having no conflict of interest, 83.3% of the conclusions were that SSB consumption could be a potential risk factor for weight gain. By contrast, the same percentage of reviews in which a potential financial conflict of interest was disclosed concluded that the scientific evidence was insufficient to support a positive association between SSB consumption and weight gain, or reported contradictory results and did not state any definitive conclusion about the association between SSB consumption and weight gain. Reviews in which a potential conflict of interest was disclosed were five times more likely to present a conclusion of no positive association between SSB consumption and weight gain than reviews that reported having no financial conflict of interest.

**What Do These Findings Mean?:**

These findings indicate that systematic reviews that reported financial conflicts of interest or sponsorship from food or drink companies were more likely to reach a conclusion of no positive association between SSB consumption and weight gain than reviews that reported having no conflicts of interest. A major limitation of this study is that it cannot assess which interpretation of the available evidence is truly accurate. For example, the scientists involved in the systematic reviews that reported having no conflict of interest may have had preexisting prejudices that affected their interpretation of their findings. However, the interests of the food industry (increased sales of their products) are very different from those of most researchers (the honest pursuit of knowledge), and recent randomized trials support a positive association between SSB consumption and overweight/obesity. Thus, these findings draw attention to possible inaccuracies in scientific evidence from research funded by the food and drink industry. They do not imply that industry sponsorship of nutrition research should be avoided entirely. Rather, as in other research areas, clear guidelines and principles (for example, sponsors should sign contracts that state that they will not be involved in the interpretation of results) need to be established to avoid dangerous conflicts of interest.

**Additional Information:**

Please access these websites via the online version of this summary at http://dx.doi.org/10.1371/journal.pmed.1001578.

- The Research Ethics Program at the University of California, San Diego provides an overview of conflicts of interest for researchers and details of US regulations and guidelines
- The *PLOS Medicine* series on Big Food examines the activities and influence of the food industry in global health
- A *PLOS Medicine* Research Article by Basu et al. uses mathematical modeling to investigate whether SSB taxation would avert obesity and diabetes in India
- A 2012 policy brief from the Yale Rudd Center for Food Policy and Obesity discusses current evidence regarding SSB taxes
- The US National Institutes of Health has regulations on financial conflicts of interest for institutions applying to receive funding
- Wikipedia has pages on conflict of interest, reporting bias, systematic review, and SSBs (note that Wikipedia is a free online encyclopedia that anyone can edit; available in several languages)

## Introduction

The use of industry funding for scientific research might influence the results of published studies, leading to conclusions that could ultimately support the industry's interests. Examples of this include biased findings by the tobacco industry [1] and the energy industry's approach to climate change [2]. This issue has been thoroughly discussed in pharmaceutical studies, where the dangers of the use of so-called scientific evidence in industry marketing and profit-making strategies have been noted [3]–[6]. However, little is known about the potential role of industry sponsorship in the field of nutrition, where not only industry-sponsored studies might be biased: it is also possible that some degree of bias might exist in studies conducted by academic authors who work independently of industry funding. They may have other sources of funding with other interests, and they may have their prejudices, too. In an ideal world free from such biases, a perfect consistency between studies with different sources of funding would be expected. If that consistency is not found, this may represent empirical evidence of bias. This issue requires serious analysis, because biased information about scientific evidence regarding nutrition may negatively affect the health of the entire population. Furthermore, scientific evidence from nutrition research leads to the formulation of governmental and professional dietary guidelines as well as public health interventions and regulation [7], heightening our concern regarding possible partiality or distortion of facts.

The influence of sugar-sweetened beverages (SSBs) on weight gain and obesity has been extensively researched and debated in the last few years [8],[9]. The potential influence of the source of sponsorship has become a crucial issue in this context, because high financial profits are at stake [10],[11]. For example, in May 2008, the Australian Competition and Consumer Commission reported that a marketing campaign for a well-known SSB company was misleading [12]. However, little information exists regarding how research funded by beverage companies or sugar industries may try to counteract detrimental findings by independent researchers, and may contribute to disseminating contradictory and inconclusive information to the scientific community and the general public.

Systematic reviews (SRs) and meta-analyses represent an efficient and comprehensive way to access the available evidence on particular exposure–disease associations. However, publication bias related to authors' conflicts of interest in a SR may affect the reliability of its conclusions. Beverage and sugar industries tend to play leading roles in the reported conflicts of interest of some researchers actively publishing in the field of SSB and obesity. Therefore, we assessed whether the disclosure of potential financial conflicts of interest with these industries was associated with conclusions on SSB consumption and weight gain or obesity in published SRs.

## Methods

Standard methods for conducting and reporting SRs were used [13].

We conducted a search of three databases (PubMed [http://www.ncbi.nlm.nih.gov/pubmed/], the Cochrane Library [http://www.thecochranelibrary.com/view/0/index.html], and Scopus [http://www.elsevier.com/online-tools/scopus]) in the time period from the inception of the databases to August 31, 2013. Additional articles were identified from reference lists of relevant studies and reviews. We used a sensitive search strategy in order to retrieve SRs of studies conducted on humans and written in English, Spanish, or French. The following combinations of terms were used: (soft drink or soft drinks or soda or beverage*) and (body mass index or bmi or weight or obes* or overweight). We included all SRs (with or without meta-analyses) that stated specific search criteria and information about the databases used, and that conducted a SR on the topic of SSBs as a potential risk factor for weight gain or obesity.

Two researchers, blinded to the authors' financial conflicts of interest and stated sources of funding, independently extracted the conclusions stated in the articles. The agreement between the researchers was 93.3% (Kappa index: 0.86; *p*<0.001); disagreement was resolved through a third researcher's assessment, to reach a consensus. Based on these conclusions, we classified the SRs into those that had found a positive association versus those that had not for the relationship between SSB consumption and weight gain or obesity. SRs were considered to have a conclusion of a positive association when they concluded that SSB consumption may increase the risk of weight gain or overweight/obesity. By contrast, SRs were considered to have a conclusion of no positive association when they concluded that there was insufficient evidence to assess the risk of SSB consumption on weight gain or obesity, or when they presented contradictory results without stating any definitive conclusion about the association.

A third researcher classified the SRs according to the authors' financial conflicts of interest or stated sources of funding. Potential financial conflicts of interest were identified if an explicit statement in this regard was made in the text by any of the authors, or if a declared affiliation or financial disclosure by the author evidenced a link with a food industry.

We also evaluated whether the identified SRs used standard methods for conducting and reporting SRs.

The association between stated conflicts of interest and the articles' conclusions was assessed using Poisson regression analysis to adjust for year of publication and whether the article was published in a journal in the first quartile of impact factor within its category (according to ISI Web of Knowledge), or to adjust for the number of citations per year for each SR since its publication. We also adjusted for the existence/inexistence of summarizing point estimates (meta-analyses). Risk ratios and their 95% confidence intervals were calculated. Stata 12.1 (StataCorp) was used for all analyses.

## Results

**Figure 1:**
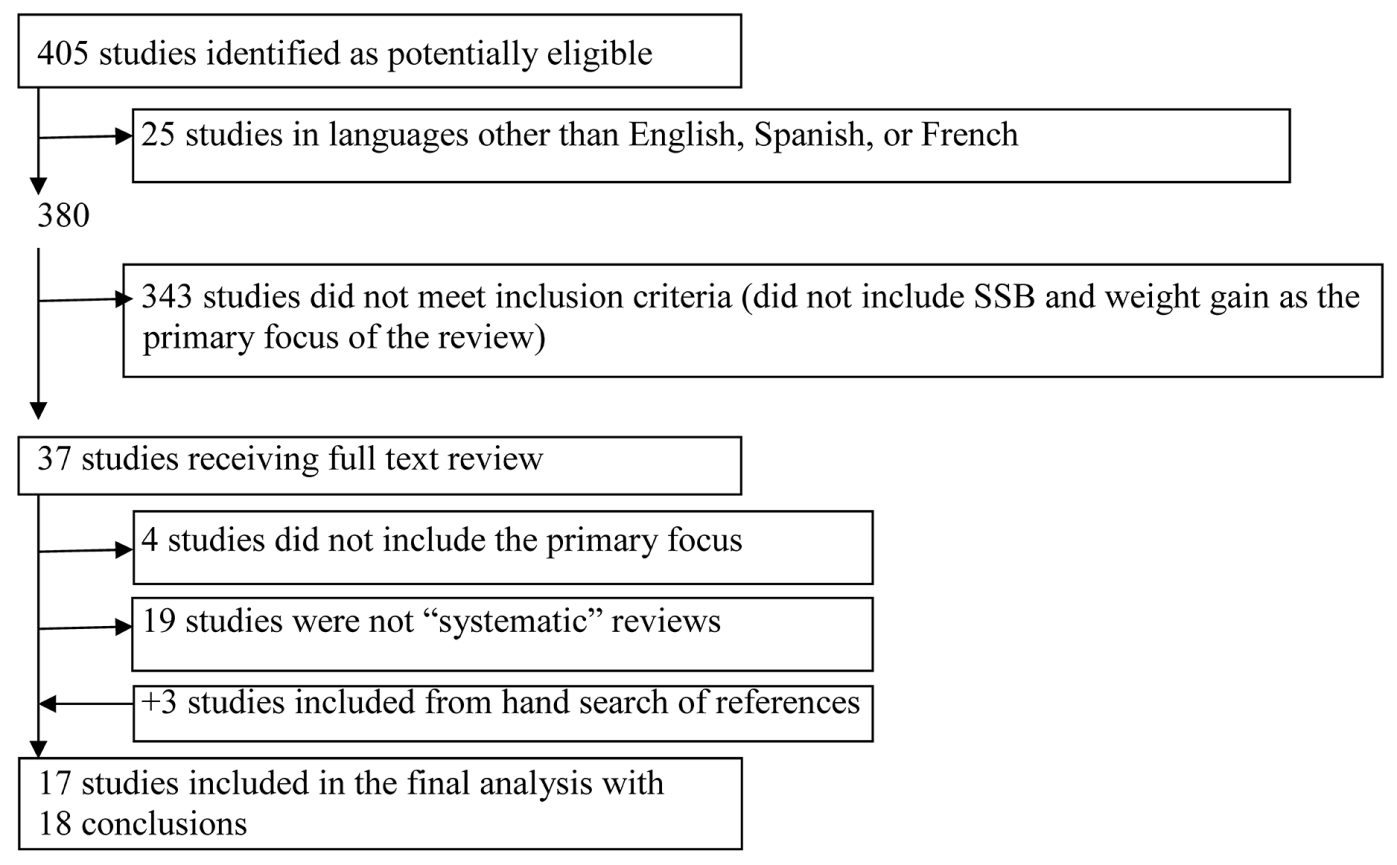
Flow-chart of systematic reviews included in the final analysis. The search used the following combinations of terms: (soft drink or soft drinks or beverage* or soda) and (body mass index or bmi or weight or obes* or overweight); filters: Review and Human; date: up to August 31, 2013.

Our final study was based on 17 out of 405 potentially eligible articles (Figure 1) [14]–[30]. Twelve of them focused on adults and child/adolescent populations, four only on children/adolescents, and one only on adults. Twelve were SRs with no meta-analysis (Table 1).

**Table 1:** Characteristics of the systematic reviews about sugar-sweetened beverages and obesity up to August 31, 2013.

SR	Year of Publication	Number of Studies Included in the SRs	Population	Study Type	Conflicts of Interest with Food Companies	Grading of SR Conclusion	SR Conclusions (Meta-Analysis Estimates)
Malik et al. [29]	2013	22 prospective studies10 RCTs	Adults and children/adolescents	SR and meta-analysis	No	Positive association	“Our systematic review and meta-analysis of prospective cohort studies and RCTs provides evidence that SSB consumption promotes weight gain in children and adults.” (Prospective studies—children: BMI change: 0.06; 95% CI: 0.02–0.10; prospective studies—adults: weight change: 0.22 kg, 95% CI: 0.09–0.34, per one daily serving increment; RCTs—children: BMI change: −0.17, 95% CI: −0.39 to 0.05, when SSBs were reduced; RCTs—adults: weight change: 0.85 kg, 95% CI: 0.50–1.20, when SSBs were added.)
Te Morenga et al. [15]	2012	7 prospective studies5 RCTs	Adults and children/adolescents	SR and meta-analysis	No	No positive association and positive association	“Trials in children which involved the recommendations to reduce intake of sugar sweetened foods and beverages showed no overall change in body weight….In prospective studies SSB consumption was associated with the risk of being overweight or obese.” (odds ratio: 1.55, 95% CI: 1.32–1.82, highest consumption versus lowest consumption).
Hauner et al. [30]	2012	23 prospective studies10 RCTs	Adults and children/adolescents	SR	No	Positive association	“The available cohort and intervention studies regarding adults mainly show that a higher consumption of sugar-sweetened beverages is accompanied by an increased risk of obesity. The overall evidence regarding an increased risk of obesity due to higher consumption of sugar-sweetened beverages in children and adolescents is therefore only judged as possible.”
Osei-Assibey et al. [14]	2012	4 prospective studies4 RCTs	Children/adolescents	SR	No	Positive association	“Providing alternatives to sugar-sweetened soft drinks should be considered in obesity prevention.”
Clabaugh and Neuberger [16]	2011	3 cross-sectional studies5 prospective studies1 RCT	Children/adolescents	SR	No	Positive association	“Education and political action by nurses to promote a decrease in SSB intake is a step in the right direction in reducing obesity in our children.”
Mattes et al. [17]	2011	12 RCTs in the SR10 RCTs in the meta-analysis	Adults and children/adolescents	SR and meta-analysis	Yes (Coca-Cola Company, PepsiCo)	No positive association	“The current evidence does not demonstrate conclusively that nutritively sweetened beverage (NSB) consumption has uniquely contributed to obesity or that reducing (NSB) consumption will reduce BMI levels in general.” (BMI change: −0.004, 95% CI: −0.079 to 0.072, when SSBs were reduced.)
Woodward-Lopez et al. [18]	2010	32 cross-sectional studies24 prospective studies	Adults and children/adolescents	SR	No	Positive association	“All lines of evidence consistently support the conclusion that the consumption of sweetened beverages has contributed to the obesity epidemic.”
Ruxton et al. [19]	2010	5 prospective studies3 RCTs	Adults and children/adolescents	SR	Yes (Sugar Bureau, UK)	No positive association	“Some studies, specifically on sweetened beverages, highlighted a potential concern in relation to obesity risk, although these were limited by important methodological issues.”
Dennis et al. [21]	2009	2 cross-sectional studies1 prospective study4 RCTs	Adults	SR	Yes (Institute for Public Health and Water Research)	Positive association	“A reduced intake of energy-containing beverages may facilitate weight management.”
Olsen and Heitmann [20]	2009	14 prospective studies5 RCTs	Adults and children/adolescents	SR	No	Positive association	“A high intake of calorically sweetened beverages can be regarded as a determinant of obesity.”
Wolff and Dansinger [25]	2008	15 cross-sectional studies10 prospective studies5 RCTs	Adults and children/adolescents	SR	No	No positive association	“Given the magnitude of the public health concern, larger and longer intervention trials should be considered to clarify the specific effects of sugar-sweetened soft drinks on body weight and other cardiovascular risk factors.”
Harrington [24]	2008	2 time trend studies3 prospective studies2 RCTs	Children/adolescents	SR	No	Positive association	“Single intervention manipulation, elimination, or marked reduction of SSB consumption may serve to decrease caloric intake, increase satiety levels, decrease tendencies towards insulin resistance, and simplify the process of weight management in children and adolescents.”
Gibson [22]	2008	23 cross-sectional studies17 prospective studies4 RCTs	Adults and children/adolescents	SR	Yes (Union of European Beverages Associations)	No positive association	“Despite the large number of studies on this topic, the inconsistencies of definition, design, statistical treatment and interpretation make it difficult to draw definitive conclusions as to whether sugar-sweetened beverages are significantly implicated in weight gain.”
Forshee et al. [23]	2008	12 (10 prospective studies and 2 RCTs) in the SR10 (8 prospective studies and 2 RCTs) in the meta-analysis	Children/adolescents	SR and meta-analysis	Yes (American Beverage Association)	No positive association	“The quantitative meta-analysis and qualitative review found that the association between sugar-sweetened beverages consumption and BMI was near zero, based on the current body of scientific evidence.” (BMI change per one serving of SSB increase: 0.03, 95% CI: −0.01 to 0.07.)
Vartanian et al. [26]	2007	12 prospective studies7 RCTs	Adults and children/adolescents	SR and meta-analysis	No	Positive association	“Recommendations to reduce population soft drink consumption are strongly supported by the available science.” (Overall average body weight effect size: 0.08, 95% CI: 0.06–0.09.)
Forshee et al. [27]	2007	4 ecological studies8 cross-sectional studies7 prospective studies1 RCT	Adults and children/adolescents	SR	Yes (Tate and Lyle)	No positive association	“Evidence from ecological studies linking high fructose corn syrup (HFCS) or ‘soft drinks’ (proxy for HFCS) with rising BMI rates is unreliable. Evidence from epidemiologic studies and randomized controlled trials is inconclusive.”
Malik et al. [28]	2006	15 cross-sectional studies10 prospective studies5 RCTs	Adults and children/adolescents	SR	No	Positive association	“Although more research is needed, sufficient evidence exists for public health strategies to discourage consumption of sugary drinks as part of a healthy lifestyle.”

BMI, body mass index.

One of the reviews [15] was identified twice because the analyses were reported separately for adults and children/adolescents and achieved different conclusions; therefore we included a total of 18 conclusions from the 17 SRs in the analysis.

Six SRs [17],[19],[21]–[23],[27] declared potential conflicts of interest with industry, and 11 declared having no potential conflicts of interest with industry or did not report on potential conflicts of interest. Among those that reported having no potential conflicts of interest, seven of them stated that grants from governments, universities, or academic research centers funded their studies. Four SRs did not report on sources of funding [16],[20],[24],[30], although two of these SRs explicitly declared having no conflicts of interest [20],[30]. We contacted the corresponding authors for the other two SRs [16],[24], who confirmed that there were no conflicts of interest when the SRs were performed.

Sixty-one percent of the conclusions from the 17 SRs (11/18 conclusions) supported an adverse association between SSB consumption and weight gain or obesity; none reported any significant benefit.

Thirty-three percent (6/18 conclusions) of the conclusions from the 17 SRs were associated with a potential financial conflict of interest. The conflict of interest in four of these six reviews (66.7%) was based on the funding to conduct the SR. However, three out of four SRs in this category stated that the food company did not play a role in the selection or methodological assessment of the included studies, nor in the interpretation of the results or conclusions reached.

Among the SRs that reported having no conflict of interest (11 SRs with 12 conclusions), 83.3% conclusions (10/12) were that SSBs were directly associated with increased weight gain or obesity. In contrast, 83.3% (5/6) of the conclusions from SRs that reported having some conflict of interest with the food industry were that there was insufficient scientific evidence to support a positive association (Table 2). The SRs with conflicts of interest were five times more likely to present a conclusion of no positive association than those without them (95% CI: 1.29–19.34 for the non-adjusted risk ratio). This association remained basically unchanged even after considering the year of publication and the impact factor quartile of the journal in the analysis (adjusted relative risk: 5.16, 95% CI: 1.30–20.48) (Table 3). No substantial modifications were noted when adjusting for the number of citations per year since publication, or the existence of summarizing point estimates (relative risk: 4.71, 95% CI: 1.22–18.13, and relative risk: 5.00, 95% CI: 1.32–18.96, respectively).

**Table 2:** Relationship between conflicts of interest with food companies and conclusions on sugar-sweetened beverage consumption and weight gain in the systematic reviews conducted up to August 31, 2013.

Conflict of Interest with Food Companies	Grading of SR Conclusion		Total
	Number (Percent) Reporting Positive Association	Number (Percent) Reporting No Positive Association	
Yes	1 (16.7%)	5 (83.3%)	6
No	10 (83.3%)	2 (16.7%)	12

One study, Te Morenga et al. [14], that reported having no conflict of interests with food companies was included twice, one for positive association (in adults) and another for no positive association (in children and adolescents).

**Table 3:** Risk ratios for the conclusion of no positive association between sugar-sweetened beverages and weight gain in the systematic reviews conducted up to August 31, 2013.

Risk Ratio	No Conflict of Interest with Food Companies	Conflict of Interest with Food Companies
Crude risk ratio (95% CI)	1 (Ref.)	5.00 (1.29–19.34)
Adjusted for year of publication (95% CI)	1 (Ref.)	4.94 (1.23–19.90)
Adjusted for year of publication and the whether published in a journal in the first impact factor quartile of its category (95% CI)	1 (Ref.)	5.16 (1.30–20.48)

We identified only two SRs [15],[29] that specifically reported using the PRISMA guidelines as the standard method for conducting and reporting the meta-analysis. Nine of the SRs were published in 2009 or before, but none of these mentioned the MOOSE guidelines, the standard reference before the PRISMA statement was issued in 2009. One SR [19] reported the use of the Scottish Intercollegiate Guidelines Network as the guideline for reporting the SR. Among the 17 SRs included in our study, only three [15],[17],[29] reported a flowchart of the study search and selection process as recommended by PRISMA.

When we analyzed the original articles included in each SR, we observed the existence of high heterogeneity in the selection of original studies to be included in the SRs. Part, but not all, of this heterogeneity could be explained by differences in inclusion criteria such as the time frame of the meta-analyses, the types of study designs to be included (there were some SRs that included only randomized controlled trials [RCTs], others selected only prospective studies and RCTs, and other SRs also included cross-sectional studies as well as prospective studies and RCTs), and the population included in the meta-analysis (in some SRs only children and adolescents, and in other SRs only adults). We identified two SRs without conflicts of interest [14],[16] and one SR funded by the food industry [19] that did not include in their search strategy all the available literature when the review was undertaken. Similarly, another food industry–funded SR [27] did not include one of the RCTs already included in a previous SR [28], and therefore available at the time of the literature search.

## Discussion

The main finding of our assessment was that those SRs with stated sponsorship or conflicts of interest with food or beverage companies were five times more likely to report a conclusion of no positive association between SSB consumption and weight gain or obesity than those reporting having no industry sponsorship or conflicts of interest. This difference could be explained by a potential bias in the design, analysis, or interpretation of the results obtained in the SRs, depending on whether the authors reported having any financial conflict of interest or not.

To our knowledge, this is the first SR that specifically examines the influence of financial conflicts of interest on the conclusions of SRs connecting SSBs and weight gain. Our results concur with those of a previous study that concluded that industry funding of nutrition-related scientific articles may bias conclusions in favor of sponsors' products [7]. The blind and independent assessment by two researchers reinforces the reliability of our data. To maintain objectivity, we did not attempt to acquire independent information about study sponsorship beyond that declared by the authors in their articles. The strengths of our study include the blinded scaling of the conclusions of SRs, and the almost unanimous agreement between the two researchers.

It might be worthwhile to further explore the implications of biased reviews, with their consequent dissemination of flawed information to public health policy makers and medical practitioners. Industry sponsorship is likely to have contributed to this detrimental bias. On the other hand, some researchers argue that industry should be allowed to also play a role because science should not be the dominion of only one segment of society. In their view, the body of literature would be poorer in both breath and scope if some segments were excluded from the research arena [31]. We agree with this view, as industry-funded research projects, large and small, comprise a large proportion of all food science and nutrition research [32],[33], but the present results remain as a reason for concern when interpreting reviews funded by food industries with vested interests in the conclusions of those reviews.

An alternative, but less likely, explanation for our results could be the potential existence of publication bias only among non-industry-funded research projects [34].

Our results showed that potential financial conflicts of interest do influence the conclusions of SRs. It could be argued that we have not assessed which interpretation is truly accurate and that the bias might be on the part of the non-industry-funded researchers because of prejudices or publication bias [34]. However, the interests of food industry (increased sales of food products) are different from a researcher's task (which, at best, is the honest pursuit of knowledge and, at worst, the advancement of her or his career). In addition, the most recently published evidence in this field [35] and a new comprehensive SR and meta-analysis [29] support a positive direct association between SSB consumption and weight gain or obesity. The two largest and best RCTs in children [36],[37] and the largest study assessing the biological plausibility of gene–SSB interactions in adults [38] also support a detrimental effect of SSB on overweight/obesity. Notably, the last review with potential conflicts of interest was conducted in 2009. After that year, research funded by the food industry on this topic centered more on methodological issues than on providing new numerical results [39].

We should consider that, in the first place, scientific endeavor should seek the truth, irrespective of financial or other interests. If other concerns influence the results of research, nutrition science as a whole is likely to suffer, partly because of incorrect information and partly through a loss of confidence in the discipline from the general public. Eventually, nutrition research itself might be at risk because perceived biases would threaten to make it irrelevant. The influence of biased reviews on policy makers and medical practitioners might also be another potential threat for public health [40],[41]. Furthermore, we must take into account the fact that the potential misleading role of the food industry on health issues is greater in developing countries than in developed ones [42].

For all these reasons, the scientific community should make special efforts to preclude funding by parties with vested interests at all levels, to sustain the credibility of nutritional science within the general population and to protect the scientific endeavors in this field. This does not imply that industry sponsorship should be avoided completely, since it serves to drive nutrition research. However, clearly implemented guidelines and principles need to be established, to avoid dangerous conflicts of interest (for example, requiring industry sponsors to sign contracts that state that they will not participate in the selection of data or methodological assessment, nor in the interpretation of the results or conclusions reached) [32]. All the stakeholders should take the necessary steps to achieve this goal. Public health organizations should play a leading role in addressing these issues, to revitalize their upstream political functions and to regain their role in society [43]. In addition, according to Moodie and colleagues, there is no evidence to support the effectiveness or safety of self-regulation and public–private partnerships to improve public health [44]. Indeed, requiring or creating the conditions that would permit the public health sector to work independently of the food industry might actually lead to more significant results, with no threat of bias.

Our results confirm the hypothesis that authors of SRs may draw their conclusions in ways consistent with their sponsors' interests [45]. Researchers working with or sponsored by the industry may be subjected to conscious or unconscious pressure, and they need help to resist such pressure. In this context, journal editors have a role in reducing this potential bias by formulating and reinforcing policies that require the disclosure of conflicts of interest, and assuring that objective standards are achieved [46]. A uniform and full disclosure of conflicts of interest is an important, though not the only, step in the prevention of bias in research, though some might consider it excessively burdensome [47].

Summarizing, conclusions that are based on evidence about SSBs and weight gain substantially differ depending on the authors' financial conflicts of interest. SRs supported by beverage and sugar industries frequently reported a lack of association between the consumption of SSBs and obesity, leading to contradictory results when compared to original studies included in the SR. However, most reviews that reported having no conflicts of interest argued that current evidence justified public health strategies that discourage the consumption of SSBs. This lack of consistency between both groups of SRs suggests an empirical evidence of bias.

### Conclusions

SRs with financial conflicts of interest were five times more likely to present a conclusion of no positive association between SSB consumption and obesity than those without them.

Our findings serve to draw attention to possible inaccuracies in scientific evidence from research funded by the food industry.

## Supporting Information

Protocol S1 **Protocol for the systematic review.**
(PDF)

Table S1 **Original articles included in the systematic reviews.**
(DOCX)

Text S1 **PRISMA statement.**
(DOCX)

## Abbreviations

RCT: randomized controlled trial
SR: systematic review
SSB: sugar-sweetened beverage
