# Supplementary material for: Financial Conflicts of Interest and Reporting Bias Regarding the Association between Sugar-Sweetened Beverages and Weight Gain: A Systematic Review of Systematic Reviews
Source: PLoS Med. 2013 Dec 31;10(12):e1001578. doi: 10.1371/journal.pmed.1001578 (PMC3876974; doi:10.1371/journal.pmed.1001578)
Supplement: Protocol S1 — Protocol for the systematic review. (PDF) [file pmed.1001578.s001.pdf]

Protocol for a Systematic Review

Title: Sugar-sweetened beverages and weight gain: is the food industry sponsorship biasing the conclusions? A systematic review.

Lead reviewer's contact details.
Maira Bes-Rastrollo
Dept. of Preventive Medicine and Public Health
School of Medicine
University of Navarra (Spain)
c/ Irunlarrea, 1 (Ed. Investigacion). 31008 Pamplona (Navarra), Spain
Tel. +34 948425600 Ext.806602
Fax. +34 948425649
E-mail: mbes@unav.es

Sugar-sweetened beverages and weight gain: is the food industry sponsorship biasing the conclusions? A systematic review.

1. Background

Industry sponsorship of biomedical research might bias scientific conclusions. This issue has been thoroughly commented in pharmaceutical studies and it can be especially worrying because of the use of so-called "scientific evidence" in industry marketing strategies for the sake of their own profits [1-4]. However, little is known about the potential role of industry sponsorship in the area of nutrition despite that bias on nutrition scientific evidence may have a negative effect on the health of the whole population. In contrast with the pharmaceutical area with selective target in the small fraction of the population who is affected by a particular disease, nutrition has a target that usually includes 100% of the population. Furthermore, scientific evidence from nutrition research leads to the formulation of governmental and professional dietary guidelines as well as public health interventions and regulation [5].

In this context, the role of sugar-sweetened beverages (SSB) on weight gain and obesity has been extensively investigated and debated during the last years [6,7]. The potential influence of the source of sponsorship is highly relevant because high financial profits are at stake [8,9]. For example, in May 2008 the Australian Competition and Consumer Commission reported that a marketing campaign of a well-known SSB company was misleading. However, there is little information regarding how research on this topic funded by beverages or sugar industries may try to counteract the findings of independent research, and may contribute to send a message of contradictory results to the scientific community.

Systematic reviews and meta-analyses provide a resource to access the available evidence regarding a particular exposure-disease association. However, publication bias related to authors' conflict of interests of a systematic review might be an important issue that may compromise the reliability of its conclusions. The beverage and sugar industries tend to be especially present in the reported conflicts of interest of some investigators actively publishing in the field of SSB and obesity. Therefore, we assessed whether the disclosure of a potential financial conflict of interest with these industries was associated with the conclusions on SSB consumption and weight gain or obesity in published meta-analyses and systematic reviews.

2. Objective of the Review

To assess whether the disclosure of a potential financial conflict of interest with food industries was associated with the conclusions on SSB consumption and weight gain or obesity in published meta-analyses and systematic reviews.

3. Methods.

Criteria for inclusion studies in the review

Published systematic reviews or meta-analyses with a specific stated search criteria and information about the used databases that conducted a systematic review on the topic of SSB as a potential risk factor for weight gain or obesity (primary focus).

Databases: PubMed and Cochrane Database. Plus, hand search references.

Time and place: Studies published at any time until 31st January 2013.

Study characteristics: Systematic reviews or meta-analyses conducted in human.

Languages: English, Spanish, and French

Search: (soft drink or soft drinks or beverage* or soda) and (body mass index or bmi or weight or obes* or overweight)

Flow diagram of study selection procedure

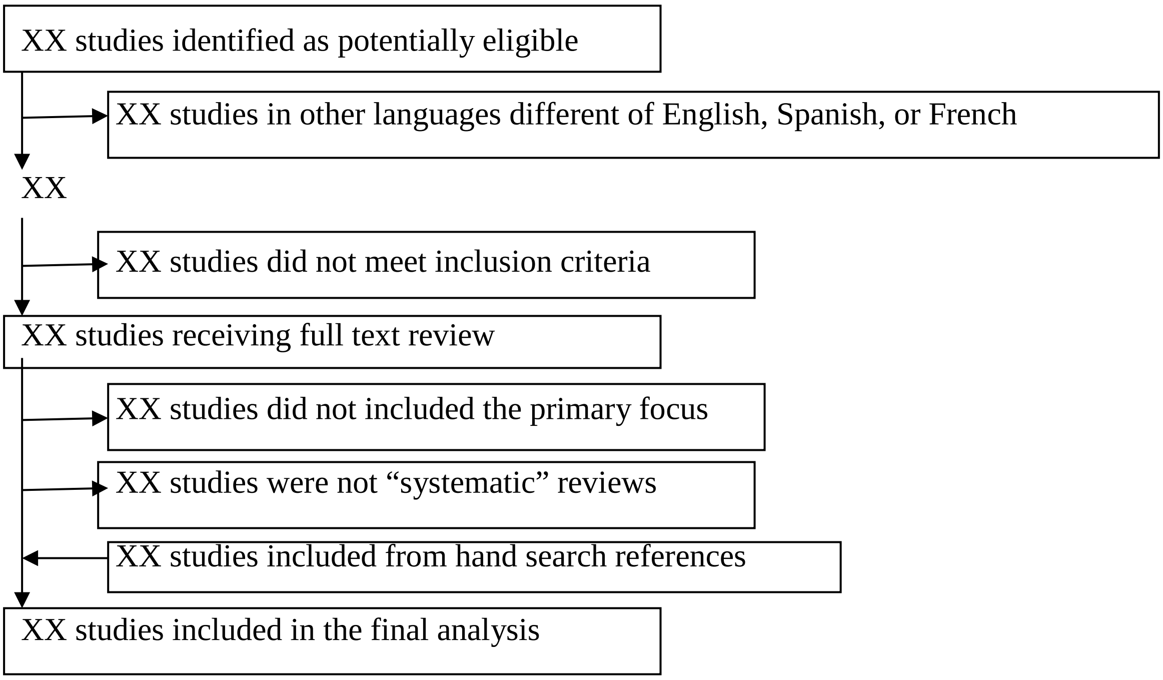

Data management: One reviewer will conduct the search strategy, will classify the systematic review or meta-analysis according to the authors' financial conflicts of interest or stated source of finding. Potential financial conflicts of interests will be identified if an explicit statement on this regard is made in the manuscript by any of the authors; or if a declared affiliation or financial disclosure by any author suggesting a link with a food industry is made. This reviewer will provide to other two reviewers the final systematic reviews or meta-analyses included in the study that will be blinded to the authors' financial conflicts of interest or stated source of funding. These two independent reviewers based on the conclusion of the systematic reviews or meta-analyses will classify them into those which found a positive or non-positive association on the relationship between SSB consumption and weight gain or obesity. Conclusions with a positive association will be considered when a systematic review concludes that SSB consumption may increase the risk of weight gain or overweight/obesity. By

contrast, conclusions with a non-positive association will be considered when the systematic review emphasizes that there is insufficient evidence to assess the risk of SSB consumption on weight gain or obesity or sent a message of contradictory results without any clear conclusion. If there is disagreement between the two researchers, the disagreement will be solved by a third reviewer reaching a consensus.

4. Timeframe

It is expected to include systematic reviews or meta-analyses up to 31st January 2013.

5. Conflicts of interest

Reviewers have no conflicts of interest to declare.

6. Funding

No funding will be received for this systematic review.

7. References

1) Bekelman JE, LiY, Gross CP (2003) Scope and impact of financial conflicts of interest in biomedical research: A systematic review. JAMA 289:454-465.
2) Kottow M (2010) Ethical quandaries posing as conflicts of interest. J Med Ethics 36:328-332.
3) Wang AT, McCoy CP, Murad MH, Montori VM (2010) Association between industry affiliation and position on cardiovascular risk with rosiglitazone: cross sectional systematic review. BMJ. doi: 10.1136/bmj.c1344.
4) Angell M (2005) The Truth About the Drug Companies: How They Deceive Us and What to Do About It. New York: Random House Trade Paperbacks.
5) Lesser LI, Ebbeling CB, Goozner M, Wypij D, Ludwig DS (2007) Relationship between funding source and conclusion among nutrition-related scientific articles. PLoS Med 4:e5. doi: 10.1371/journal.pmed.0040005.
6) Allison DB, Mattes RD (2009) Nutritively sweetened beverage consumption and obesity: the need for solid evidence on a fluid issue. JAMA 301:318-320.
7) Weed DL, Althuis MD, Mink PJ (2011) Quality of reviews on sugar-sweetened beverages and health outcomes: a systematic review. Am J Clin Nutr 94:1340-1347.
8) Nestle M (2007) Food Politics: How the Food Industry Influences Nutrition and Health. 2nd ed. Berkeley: University of California Press.
9) Nestle M (2001) Food company sponsorship of nutrition research and professional activities: a conflict of interest? Public Health Nutr 4:1015-1022.
