# Supplementary material for: Financial Conflicts of Interest and Reporting Bias Regarding the Association between Sugar-Sweetened Beverages and Weight Gain: A Systematic Review of Systematic Reviews
Source: PLoS Med. 2013 Dec 31;10(12):e1001578. doi: 10.1371/journal.pmed.1001578 (PMC3876974; doi:10.1371/journal.pmed.1001578)
Supplement: Table S1 — Original articles included in the systematic reviews. (DOCX) [file pmed.1001578.s002.docx]

|  | **Malik, 2013** | **Te Morenga,**  **2012** | **Hauner, 2012** | **Osei-Assibey, 2012** | **Clabaugh, 2011** | **Mattes, 2011*** | **Woodward-Lopez, 2010** | **Ruxton, 2010*** | **Dennis,**  **2009*** | **Olsen, 2009** | **Wolff, 2008** | **Harrington, 2008** | **Gibson, 2008*** | **Forshee, 2008*** | **Vartanian, 2007** | **Forshee, 2007*** | **Malik, 2006** |
| --- | --- | --- | --- | --- | --- | --- | --- | --- | --- | --- | --- | --- | --- | --- | --- | --- | --- |
| **Cites**** | NA | 7 | 10 | 0 | 3 | 21 | 7 | 31 | 26 | 35 | 26 | 41 | 55 | 102 | 359 | 53 | 608 |
| **Cross-sectional in children** |  |  |  |  |  |  |  |  |  |  |  |  |  |  |  |  |  |
| Gilbert, 1992 |  |  |  |  |  |  |  |  |  |  |  |  |  |  | X |  |  |
| Bandini, 1999 |  |  |  |  |  |  |  |  |  |  | X |  | X |  |  |  | X |
| Gibson, 1999 |  |  |  |  |  |  |  |  |  |  | X |  | X |  |  |  | X |
| Troiano, 2000 |  |  |  |  |  |  |  |  |  |  | X |  | X |  |  |  | X |
| Zizza, 2001 |  |  |  |  |  |  |  |  |  |  |  |  |  |  |  | X |  |
| French, 2003 |  |  |  |  |  |  |  |  |  |  |  |  |  |  |  | X | X |
| Forshee, 2003 |  |  |  |  |  |  |  |  |  |  |  |  | X |  | X | X | X |
| Giammattei, 2003 |  |  |  |  | X |  |  |  |  |  | X |  | X |  | X | X | X |
| Gillis, 2003 |  |  |  |  | X |  |  |  |  |  | X |  | X |  | X |  | X |
| McGartland, 2003 |  |  |  |  |  |  |  |  |  |  |  |  |  |  | X |  |  |
| Nicklas, 2003 |  |  |  |  |  |  |  |  |  |  |  |  | X |  | X | X | X |
| Rodriguez-Artalejo, 2003 |  |  |  |  |  |  |  |  |  |  | X |  | X |  | X |  | X |
| Ariza, 2004 |  |  |  |  |  |  |  |  |  |  | X |  | X |  | X |  |  |
| Cullen, 2004 |  |  |  |  |  |  |  |  |  |  |  |  |  |  | X |  |  |
| Forshee, 2004 |  |  |  |  |  |  |  |  |  |  | X |  | X |  | X | X | X |
| Grant, 2004 |  |  |  |  |  |  |  |  |  |  |  |  |  |  |  | X |  |
| Novotny, 2004 |  |  |  |  |  |  |  |  |  |  |  |  | X |  | X |  |  |
| Overby, 2004 |  |  |  |  |  |  |  |  |  |  | X |  |  |  |  |  | X |
| Andersen, 2005 |  |  |  |  |  |  |  |  |  |  | X |  | X |  |  |  | X |
| Forshee, 2005 |  |  |  |  |  |  |  |  |  |  |  |  |  |  | X |  |  |
| Jansen, 2005 |  |  |  |  |  |  |  |  |  |  |  |  | X |  |  |  |  |
| Rajeshwari, 2005 |  |  |  |  | X |  |  |  |  |  |  |  | X |  | X | X |  |
| Veugelers, 2005 |  |  |  |  |  |  |  |  |  |  |  |  | X |  |  |  |  |
|  | **Malik, 2013** | **Te Morenga,**  **2012** | **Hauner, 2012** | **Osei-Assibey, 2012** | **Clabaugh, 2011** | **Mattes, 2011** | **Woodward-Lopez, 2010** | **Ruxton, 2010** | **Dennis,**  **2009** | **Olsen, 2009** | **Wolff, 2008** | **Harrington, 2008** | **Gibson, 2008** | **Forshee, 2008** | **Vartanian, 2007** | **Forshee, 2007** | **Malik, 2006** |
| O’Connor, 2006 |  |  |  |  |  |  |  |  |  |  |  |  | X |  |  |  |  |
| Silveira, 2006 |  |  |  |  |  |  |  |  |  |  |  |  | X |  |  |  |  |
| Gibson, 2007 |  |  |  |  |  |  |  |  |  |  |  |  | X |  |  |  |  |
| Roseman, 2007 |  |  |  |  |  |  |  |  |  |  |  |  | X |  |  |  |  |
| Fox, 2009 |  |  |  |  | X |  |  |  |  |  |  |  |  |  |  |  |  |
| **Cross-sectional in adults** |  |  |  |  |  |  |  |  |  |  |  |  |  |  |  |  |  |
| French, 1994 |  |  |  |  |  |  |  |  |  |  | X |  | X |  |  |  |  |
| Kim, 1997 |  |  |  |  |  |  |  |  |  |  |  |  |  |  | X |  |  |
| Liebman, 2003 |  |  |  |  |  |  |  |  |  |  | X |  | X |  | X |  | X |
| Lin, 2004 |  |  |  |  |  |  |  |  | X |  |  |  |  |  |  |  |  |
| McCarthy, 2006 |  |  |  |  |  |  |  |  |  |  |  |  | X |  |  |  |  |
| Warner, 2006 |  |  |  |  |  |  |  |  |  |  |  |  | X |  |  |  |  |
| Sun, 2007 |  |  |  |  |  |  |  |  |  |  |  |  | X |  |  |  |  |
| **Prospective cohorts in children** |  |  |  |  |  |  |  |  |  |  |  |  |  |  |  |  |  |
| Alexy, 1999 |  |  |  | X |  |  |  |  |  |  |  |  |  |  |  |  |  |
| Ludwig, 2001 | X | X | X |  | X |  |  | X |  | X | :X | X | X | X | X | X | X |
| Skinner, 2001 0/- (FJ) |  | X |  |  |  |  |  |  |  |  |  |  |  |  |  |  |  |
| Mrdjenovic, 2003 | S |  | X | X |  |  |  |  |  | X |  | X | X | X | X | X |  |
| Nicklas, 2003 |  |  |  |  |  |  |  | X |  |  | X |  |  |  |  |  |  |
| Berkey, 2004 | X | X | X |  | X |  |  |  |  | X | X | X | X | X | X | X | X |
| Field, 2004 |  |  |  |  |  |  |  |  |  |  |  |  |  |  |  | X |  |
| Newby, 2004 | X |  | X |  |  |  |  |  |  | X | X |  | X | X | X | X | X |
| Philips, 2004 |  | X | X |  |  |  |  |  |  | X | X |  | X | X | X |  | X |
|  | **Malik, 2013** | **Te Morenga,**  **2012** | **Hauner, 2012** | **Osei-Assibey, 2012** | **Clabaugh, 2011** | **Mattes, 2011** | **Woodward-Lopez, 2010** | **Ruxton, 2010** | **Dennis,**  **2009** | **Olsen, 2009** | **Wolff, 2008** | **Harrington, 2008** | **Gibson, 2008** | **Forshee, 2008** | **Vartanian, 2007** | **Forshee, 2007** | **Malik, 2006** |
| Blum, 2005 | X | X | X |  | X |  |  |  |  | X | X |  | X | X | X |  | X |
| Welsh, 2005 | S | X |  | X |  |  |  | X |  | X | X |  | X |  |  |  | X |
| Faith, 2006 |  | X |  |  |  |  |  |  |  |  |  |  |  |  |  |  |  |
| Halkjaer, 2006 |  | X |  |  |  |  |  |  |  |  |  |  |  |  |  |  |  |
| Mundt, 2006 | X |  | X |  |  |  |  |  |  |  |  |  | X | X |  |  |  |
| Striegal-Moore, 2006 | X | X | X |  |  |  |  |  |  | X |  |  | X | X | X |  |  |
| Viner, 2006 | X | X |  |  |  |  |  |  |  | X |  |  |  |  |  |  |  |
| Tam, 2006 | S |  | X |  |  |  |  |  |  | X |  |  |  |  |  |  |  |
| Dubois, 2007 | S | X | X |  |  |  |  |  |  |  |  |  |  |  |  |  |  |
| Johnson, 2007 | X | X | X |  |  |  |  |  |  |  |  |  | X |  |  |  |  |
| Kral, 2008 | S |  | X |  |  |  |  |  |  |  |  |  |  |  |  |  |  |
| Laurson, 2008 | X |  |  |  |  |  |  |  |  |  |  |  |  |  |  |  |  |
| Libuda, 2008 | X | X | X |  |  |  |  |  |  |  |  |  | X |  |  |  |  |
| Williams, 2008 |  | X |  |  |  |  |  |  |  |  |  |  |  |  |  |  |  |
| Nissinen, 2009 |  | X |  |  |  |  |  |  |  |  |  |  |  |  |  |  |  |
| Fiorito, 2009 | S | X | X |  |  |  |  |  |  |  |  |  |  |  |  |  |  |
| Lim, 2009 | S | X | X |  |  |  |  |  |  |  |  |  |  |  |  |  |  |
| Vanselow, 2009 | X | X | X |  |  |  |  |  |  |  |  |  |  |  |  |  |  |
| Halkjaer, 2009 |  | X |  |  |  |  |  |  |  |  |  |  |  |  |  |  |  |
| Olsen, 2010 (unpublished) |  |  |  |  |  |  |  |  |  | X |  |  |  |  |  |  |  |
| Haerens, 2010 0 |  | X |  |  |  |  |  |  |  |  |  |  |  |  |  |  |  |
| Herbst, 2011 |  | X |  |  |  |  |  |  |  |  |  |  |  |  |  |  |  |
|  | **Malik, 2013** | **Te Morenga,**  **2012** | **Hauner, 2012** | **Osei-Assibey, 2012** | **Clabaugh, 2011** | **Mattes, 2011** | **Woodward-Lopez, 2010** | **Ruxton, 2010** | **Dennis,**  **2009** | **Olsen, 2009** | **Wolff, 2008** | **Harrington, 2008** | **Gibson, 2008** | **Forshee, 2008** | **Vartanian, 2007** | **Forshee, 2007** | **Malik, 2006** |
| Stoff, 2011 + |  | X |  |  |  |  |  |  |  |  |  |  |  |  |  |  |  |
| Weijs, 2011 + |  | X |  |  |  |  |  |  |  |  |  |  |  |  |  |  |  |
| Carlson, 2012 | X |  |  |  |  |  |  |  |  |  |  |  |  |  |  |  |  |
| Laska, 2012 | X |  |  |  |  |  |  |  |  |  |  |  |  |  |  |  |  |
| Olsen, 2012 | X |  |  |  |  |  |  |  |  |  |  |  |  |  |  |  |  |
| **Prospective cohorts in adults** |  |  |  |  |  |  |  |  |  |  |  |  |  |  |  |  |  |
| French, 1994 | X | X |  |  |  |  |  |  |  |  | X |  | X |  |  |  | X |
| Schulz, 2002 |  | X |  |  |  |  |  |  |  | X |  |  | X |  |  |  |  |
| Janket, 2003 |  |  |  |  |  |  |  |  |  |  |  |  |  |  |  | X |  |
| Schulze, 2004 | X | X | X |  |  |  |  | X |  | X | X |  | X |  | X | X | X |
| Drapeau, 2004 |  | X |  |  |  |  |  |  |  |  |  |  |  |  | X |  |  |
| Kvaavik, 2005 | S | X | X |  |  |  |  | X | X | X | X |  | X |  | X |  | X |
| Nooyens, 2005 | X | X | X |  |  |  |  |  |  |  |  |  | X |  |  |  |  |
| Bes-Rastrollo, 2006 | S | X | X |  |  |  |  |  |  | X | X |  | X |  | X |  | X |
| Dhingra, 2007 | S | X | X |  |  |  |  |  | X |  |  |  |  |  |  |  |  |
| Palmer, 2008 | X | X |  |  |  |  |  |  |  |  |  |  |  |  |  |  |  |
| Stookey, 2008 | X |  |  |  |  |  |  |  |  |  |  |  |  |  |  |  |  |
| Nissinen, 2009 |  |  | X |  |  |  |  |  |  |  |  |  |  |  |  |  |  |
| Chen, 2009 | X |  | X |  |  |  |  |  |  |  |  |  |  |  |  |  |  |
| Odegaard, 2010 | S | X |  |  |  |  |  |  |  |  |  |  |  |  |  |  |  |
| Mozaffarian, 2011 | X | X |  |  |  |  |  |  |  |  |  |  |  |  |  |  |  |
| Barone Gibbs, 2012 | X |  |  |  |  |  |  |  |  |  |  |  |  |  |  |  |  |
|  | **Malik, 2013** | **Te Morenga,**  **2012** | **Hauner, 2012** | **Osei-Assibey, 2012** | **Clabaugh, 2011** | **Mattes, 2011** | **Woodward-Lopez, 2010** | **Ruxton, 2010** | **Dennis,**  **2009** | **Olsen, 2009** | **Wolff, 2008** | **Harrington, 2008** | **Gibson, 2008** | **Forshee, 2008** | **Vartanian, 2007** | **Forshee, 2007** | **Malik, 2006** |
| **RCT in children** |  |  |  |  |  |  |  |  |  |  |  |  |  |  |  |  |  |
| Teufel, 1998 |  |  |  |  |  |  | X |  |  |  |  |  |  |  |  |  |  |
| Beech, 2003 |  |  |  |  |  |  | X |  |  |  |  |  |  |  |  |  |  |
| James, 2004 | X | X | X | X |  | X | X | X |  | X | X | X | X | X | X | X | X |
| Ebbeling, 2006 | X | X | X |  | X | X |  | X |  | X | X | X | X | X | X |  | X |
| Muñoz, 2006 |  |  | X |  |  | X |  |  |  |  |  |  |  |  |  |  |  |
| James, 2007 | S |  |  |  | X | X |  |  |  |  |  |  |  |  |  |  |  |
| Williams, 2007 |  |  |  |  |  | X |  |  |  |  |  |  |  |  |  |  |  |
| Albala, 2008 | S |  | X |  |  | X |  |  |  |  |  |  |  |  |  |  |  |
| Jordan, 2008 |  |  |  |  |  |  | X |  |  |  |  |  |  |  |  |  |  |
| Mckelbauer, 2009 |  |  |  | X |  |  |  |  |  |  |  |  |  |  |  |  |  |
| Sichieri, 2009 | X | X | X |  |  | X |  |  |  |  |  |  | X |  |  |  |  |
| Karanja, 2010 |  |  |  | X |  |  |  |  |  |  |  |  |  |  |  |  |  |
| De Ruyter, 2012 | X |  |  |  |  |  |  |  |  |  |  |  |  |  |  |  |  |
| Ebbeling, 2012 | X |  |  |  |  |  |  |  |  |  |  |  |  |  |  |  |  |
| **RCT in adults** |  |  |  |  |  |  |  |  |  |  |  |  |  |  |  |  |  |
| Addington, 1988 |  |  |  |  |  | X |  |  |  |  |  |  |  |  |  |  |  |
| Rodin, 1990 |  |  |  |  |  |  |  |  | X |  |  |  |  |  |  |  |  |
| Tordoff, 1990 | X | X | X |  |  | X | X |  | X | X | X |  |  |  | X |  | X |
| Mattes, 1996 |  |  |  |  |  |  |  |  | X |  |  |  |  |  |  |  |  |
| Canty, 1991 |  |  |  |  |  |  |  |  | X |  |  |  |  |  |  |  |  |
| Holt, 2000 |  |  |  |  |  |  |  |  | X |  |  |  |  |  |  |  |  |
| Di Mieglio, 2000 |  |  | X |  |  | X | X |  |  | X | X |  |  |  | X |  | X |
| Grandejean, 2000 |  |  | X |  |  | S |  |  |  |  |  |  |  |  | X |  |  |
|  | **Malik, 2013** | **Te Morenga,**  **2012** | **Hauner, 2012** | **Osei-Assibey, 2012** | **Clabaugh, 2011** | **Mattes, 2011** | **Woodward-Lopez, 2010** | **Ruxton, 2010** | **Dennis,**  **2009** | **Olsen, 2009** | **Wolff, 2008** | **Harrington, 2008** | **Gibson, 2008** | **Forshee, 2008** | **Vartanian, 2007** | **Forshee, 2007** | **Malik, 2006** |
| Buemann, 2002 |  |  |  |  |  |  |  |  | X |  |  |  |  |  |  |  |  |
| Raben, 2002 | S |  | X |  |  | S | X | X | X | X | X |  | X |  | X |  | X |
| St. Onge, 2004 |  |  |  |  |  |  |  |  | X |  |  |  |  |  |  |  |  |
| Van Wymelbeke, 2004 |  |  | X |  |  | S |  |  |  |  |  |  |  |  | X |  |  |
| Haub, 2005 |  |  |  |  |  | X |  |  |  |  |  |  |  |  |  |  |  |
| Della Valle, 2005 |  |  |  |  |  |  |  |  | X |  |  |  |  |  |  |  |  |
| Harper, 2007 |  |  |  |  |  |  |  |  | X |  |  |  |  |  |  |  |  |
| Melanson, 2007 |  |  |  |  |  |  |  |  | X |  |  |  |  |  |  |  |  |
| Monsivais, 2007 |  |  |  |  |  |  |  |  | X |  |  |  |  |  |  |  |  |
| Reid, 2007 | X | X |  |  |  | X | X |  |  |  |  |  |  |  |  |  |  |
| Reid, 2010 | X | X |  |  |  |  |  |  |  |  |  |  |  |  |  |  |  |
| Aeberli, 2011 | X | X |  |  |  |  |  |  |  |  |  |  |  |  |  |  |  |
| Maersk, 2012 | X |  |  |  |  |  |  |  |  |  |  |  |  |  |  |  |  |

*Existence of potential financial conflicts of interest

**Total number of cites according to ISI Web of Knowledge

X: study identified

S: study identified but excluded of the meta-analysis for methodological reasons.

NA: Not Applicable
